# The HECT E3 Ligase E6AP/UBE3A as a Therapeutic Target in Cancer and Neurological Disorders

**DOI:** 10.3390/cancers12082108

**Published:** 2020-07-29

**Authors:** Asia Owais, Rama K. Mishra, Hiroaki Kiyokawa

**Affiliations:** 1 Department of Pharmacology, Northwestern University, Chicago, IL 60611, USA; asiaowais2018@u.northwestern.edu; 2 Department of Biochemistry and Molecular Genetics, Northwestern University, Chicago, IL 60611, USA; r-mishra@northwestern.edu; 3 Center for Molecular Innovation and Drug Discovery, Northwestern University, Evanston, IL 60201, USA

**Keywords:** ubiquitin, viral oncogenesis, oncoproteins, tumor suppressors, Angelman syndrome, autism, small molecules, imprinting, E3 ligase, chromosome 15q

## Abstract

The HECT (Homologous to the E6-AP Carboxyl Terminus)-family protein E6AP (E6-associated protein), encoded by the *UBE3A* gene, is a multifaceted ubiquitin ligase that controls diverse signaling pathways involved in cancer and neurological disorders. The oncogenic role of E6AP in papillomavirus-induced cancers is well known, with its action to trigger p53 degradation in complex with the E6 viral oncoprotein. However, the roles of E6AP in non-viral cancers remain poorly defined. It is well established that loss-of-function alterations of the *UBE3A* gene cause Angelman syndrome, a severe neurodevelopmental disorder with autosomal dominant inheritance modified by genomic imprinting on chromosome 15q. Moreover, excess dosage of the *UBE3A* gene markedly increases the penetrance of autism spectrum disorders, suggesting that the expression level of UBE3A must be regulated tightly within a physiologically tolerated range during brain development. In this review, current the knowledge about the substrates of E6AP-mediated ubiquitination and their functions in cancer and neurological disorders is discussed, alongside with the ongoing efforts to pharmacologically modulate this ubiquitin ligase as a promising therapeutic target.

## 1. Introduction and E6AP Structure

Ubiquitination is a major posttranslational modification which governs the fates of modified protein substrates, i.e., changes in stability, subcellular localization and enzymatic activities [1]. The multi-step process of ubiquitination involves the activation, conjugation and ligation of a ubiquitin moiety mediated by E1, E2, and E3 enzymes, respectively. E3 ligases play crucial roles in recruiting substrates and they primarily determine substrate specificity [1]. Aberrant regulation of E3 ligases disrupts diverse cellular functions such as cell cycle control, DNA damage response and cell death, and could shift the balance towards oncogenesis [2]. The human genome encodes more than 600 E3 ligases, which are classified into a few major families based on their structural similarities: RING (Really Interesting New Gene), RBR (ring between ring), Ubox and HECT E3 ligases [3]. In this review, we focus on one of the HECT E3 ligases, E6AP, which is also known as UBE3A and plays roles in oncogenesis, neurodevelopmental disorders, and other human diseases [4].

**Figure 1:**
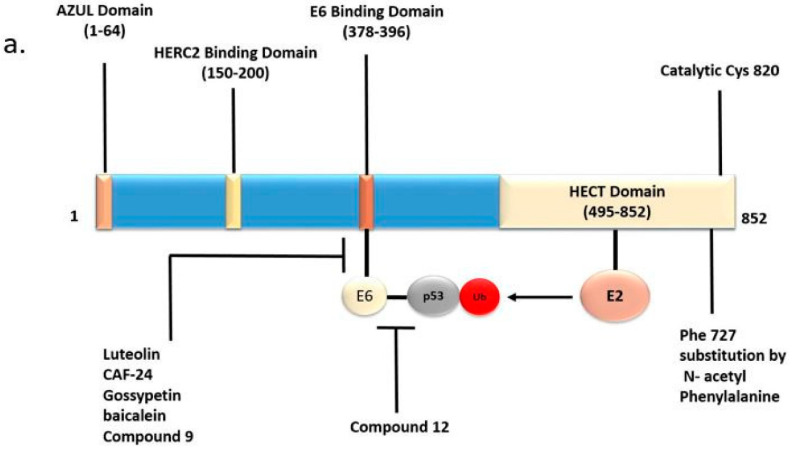

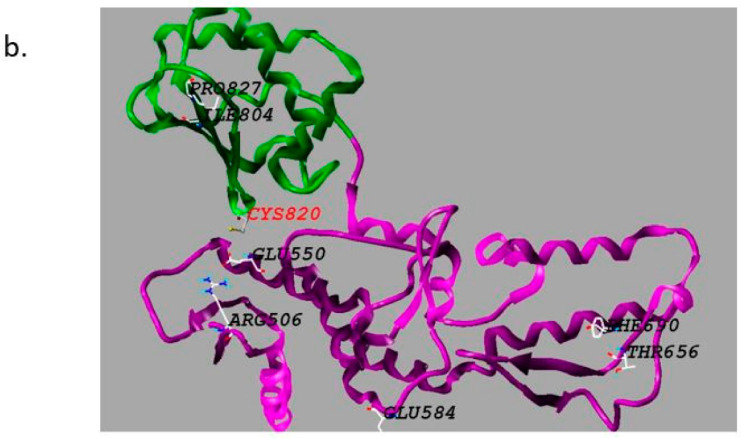
E6AP domains and their therapeutic significance. (**a**) Domains in the primary sequence of E6AP protein. The numbers in parentheses indicate the corresponding amino acid residues of E6AP isoform 1 protein. The C-terminus of E6AP protein has the prototype Homologous to the E6-AP Carboxyl Terminus (HECT) domain. This domain has the E2-binding site and the catalytic center Cys820. E6-binding domain binds the HPV E6 oncoprotein. The Zn-binding Amino-terminal Zn-finger of Ube3a Ligase (AZUL) domain is present at the N terminus. The HERC2-binding domain is required for the association with the partner HECT E3 HERC2. Small molecule inhibitors targeting the E6AP-E6 and E6-p53 interactions that disrupt the E6AP-E6-p53 complex are potential therapeutic strategies for human papillomavirus (HPV). Substitution of Phe727 by N-acetyl phenylalanine inhibits the oligomerization of E6AP essential for its activity. (**b**) Crystal structure of the HECT domain and the locations of Angelman syndrome (AS)-linked missense mutations. The N lobe is colored in magenta and the C lobe is colored in green. The catalytic Cys820 is labeled in red. Hereditary and de novo missense mutations found in the indicated amino acids are thought to be pathogenic in AS patients. The Biopolymer module of Tripos software was used to generate the figure, considering the E6AP part of 1C4z.pdb crystal structure.

HECT-E3s ubiquitinate their specific substrate in a two-step process. First, an HECT-E3 binds to an E2 in complex with activated ubiquitin, leading to the formation of a thioester linkage between the C-terminus of ubiquitin and the catalytic cysteine residue in the HECT domain. This transient complex subsequently transfers ubiquitin to an interacting substrate with the formation of an isopeptide bond [5]. Structurally, E6AP possesses a Zn^2+^-binding N-terminal (Amino-terminal Zn-finger of Ube3a Ligase (AZUL)) domain and a catalytic HECT domain of ~350 amino acids at the C terminus (Figure 1a) [6]. A domain necessary for binding with the human papillomavirus (HPV) E6 oncoprotein is located between the AZUL and HECT domains [7]. The AZUL domain is involved in substrate recruitment and also self-inhibitory regulation [8]. Most mutations associated with the neurodevelopmental disorder Angelman syndrome (AS) are found in the HECT domain, indicating that the loss of catalytic function of E6AP causes AS [9]. Considering the human E6AP-UBCH7 complex crystal structure (1C4Z.pdb) [6], we have extracted out the E6AP part by the Biopolymer module implemented in Tripos software and highlighted AS-associated mutations and the catalytic Cys820 residue (Figure 1b). The N lobe (labeled in magenta) binds to the specific E2 partner, UBE2L3/UbcH7. Many AS mutation sites are located in the areas on both the C lobe (labeled in green) and N lobe surrounding Cys820, while some mutations are found in the E2-binding region of the N lobe, e.g., Thr656 and Phe690 [10]. AS-linked missense mutations have also been reported in the AZUL domain [9,11], suggesting the significance of the domain in catalytic regulation. Aside from its ubiquitin ligase function, E6AP also acts as a transcriptional co-activator of steroid hormone receptors such as estrogen, progesterone and androgen receptors (ER, PR and AR, respectively) [4,12,13]. E6AP contains a nuclear localization signal that allows it to be localized to the nucleus, and three LxxL motifs, which are important for receptor interaction [4,14,15]. The transactivating function of E6AP has been mapped to a region outside the HECT domain (residues 170–680) [4].

Recent evidence suggests that E6AP exists in two conformational states of high and low activity. While the potent activation of E6AP by E6 binding is well known, the binding with HERC2, another HECT E3, also stimulates the activity of E6AP (Figure 1a) [16,17]. Mutations in HERC2 which cause degradation of HERC2 have been associated with an AS-like syndrome. Furthermore, protein kinase A-mediated phosphorylation of Thr485, which is located outside the HECT domain, inhibits E6AP activity. An autism-linked missense mutation that disrupts this phosphorylation site causes enhanced E6AP activity [18].

## 2. E6AP in Cancer

The discovery of E6AP lends itself to the seminal finding that the human papillomavirus (HPV) oncoprotein E6 mediates degradation of the tumor suppressor protein p53 by activating this E3 [19], which is a classic example of the downregulation of tumor suppressors by viral oncoproteins. E6AP has been studied for its involvement in not only viral oncogenesis but also non-viral oncogenesis [20,21,22,23], as recently reviewed by Bandilovska et al. [24]. Here, we focus on the roles of diverse targets of E6AP in various cancers, as summarized in Table 1.

### 2.1. HPV-Associated Cancers

HPV induces a variety of human cancers such as cervical and head and neck cancers. The involvement of E6AP in viral oncogenesis was first established in HPV-associated cervical cancer [19,25]. The HPV oncoprotein E6 hijacks the E3 activity of E6AP by acting as an allosteric activator, and mediates polyubiquitination and the subsequent proteasomal degradation of p53 [19,26]. On the other hand, the HPV oncoprotein E7 binds to the tumor suppressor retinoblastoma protein (Rb) and converts the associated E2F transcription factors from repressors to transactivators [27]. Thus, HPV infection effectively targets the two major tumor suppressors p53 and Rb in epithelial cells, leading to malignant transformation and cancer development.

### 2.2. HCV-Associated Cancers

Chronic inflammation from hepatitis C virus (HCV) infection leads to hepatocellular carcinoma [28]. E6AP promotes HCV-induced oncogenesis by mediating the proteasomal degradation of Rb protein. The RNA-dependent RNA polymerase of HCV, non-structural protein 5B (NS5B), sequesters Rb in the cytoplasm where E6AP ubiquitinates and degrades Rb [29]. E6AP also plays a role in antiviral defense by ubiquitinating and degrading a core HCV protein [30]. In turn, HCV suppresses E6AP expression by DNA methylation [31]. Thus, E6AP appears to activate antiviral defense, while it promotes oncogenic transformation induced by HCV.

### 2.3. Cancers Associated with PML Downregulation

**Figure 2:**
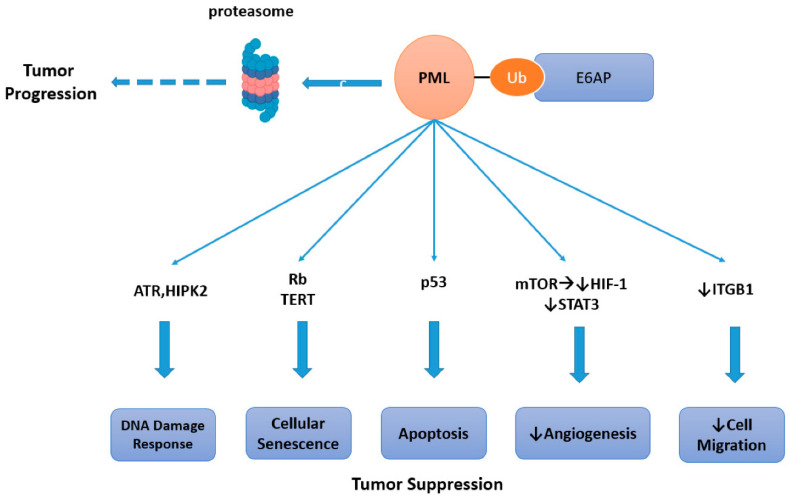
E6AP ubiquitinates promyelocytic leukemia protein (PML) and leads to tumor progression. PML causes tumor suppression by regulating cellular apoptosis [38], DNA damage response [42] and cellular senescence [46,50]. It inhibits angiogenesis and cell migration [37,44,49]. E6AP-mediated ubiquitination of PML leads to its proteasomal degradation and promotes tumorigenesis [38]. ATR-Ataxia telangiectasia and Rad3-related protein kinase, HIPK2-Homeodomain Interacting Protein Kinase 2, TERT- telomerase reverse transcriptase, mTOR-mammalian target of rapamycin, HIF-1-hypoxia inducible factor.

The tumor suppressor promyelocytic leukemia protein (PML) has been identified as a ubiquitination target of E6AP. E6AP-mediated degradation of PML promotes tumorigenesis in multiple types of cancer (Figure 2) [20,32]. *PML* was first identified as a gene fused with the retinoic acid receptor (*RAR*) gene in acute promyelocytic leukemia patients with (15; 17) chromosomal translocation. The fusion gene product suppresses the wild type tumor suppressor PML in a dominant negative fashion [33,34]. Furthermore, perturbed regulation of PML has been found in a variety of cancers without chromosomal translocations [35]. PML forms distinct structures in the nucleoplasm called the PML-nuclear body (NB). PML-NB mediates post-translational modifications of a variety of proteins, most notably conjugation with the ubiquitin-like protein Small Ubiquitin Like Modifiers (SUMO) [36]. The PML-NB clients regulate diverse cellular processes including gene transcription, DNA repair, apoptosis, senescence, and anti-viral response (Figure 2) [32,33,34,35,37,38,39,40,41,42,43,44,45,46,47,48,49,50]. Among the well-studied downstream effectors of PML are p53, Rb, signal transducer and activator of transcription 3 (STAT3) and integrin subunit beta 1 (ITGB1) [38,44,47,49,50].

#### 2.3.1. Burkitt’s Lymphoma

Hyperactivation of the E3 ligase activity of E6AP is evident in Epstein–Barr virus-associated Burkitt’s lymphoma. Approximately 60% of Burkitt’s lymphomas show downregulation of PML expression correlated with high levels of E6AP [20]. Hemizygous disruption of the *Ube3a* gene can attenuate murine B-cell lymphomagenesis driven by the Eμ (immunoglobulin enhancer)-Myc transgene with concomitant upregulation of PML [20], recapitulating the oncogenic role of E6AP in Burkitt’s lymphoma.

#### 2.3.2. Prostate Cancer

E6AP functions as a transcriptional co-activator of AR, and plays an oncogenic role in prostate glands [4,51]. E6AP expression is upregulated in a subset of prostate cancers. The oncogenic role of E6AP in prostate cancer is also associated with ubiquitin-dependent degradation of the tumor suppressor proteins PML and p27^Kip1^, which is characteristic in aggressive and late stages of prostate cancer [21,52]. A recent study that evaluated the transcriptome-and proteome-wide effects of E6AP knockdown identified new transcriptional and posttranscriptional targets of E6AP in prostate cancer [53]. Among the targets negatively regulated by E6AP is the stress-induced chaperone clusterin (CLU), which may play a tumor-suppressive role in prostate cancer.

### 2.4. Non-Small Cell Lung Cancer

In non-small cell lung cancer (NSCLC), E6AP may exert a tumor suppressive function [22]. E6AP expression is decreased in ~20% of NSCLC tissues, which correlates with lower levels of the cyclin-dependent kinase (CDK) inhibitor p16^INK4A^. E6AP binds to the transcription factor E2F1 as a co-factor, repressing the expression of CDC6, a key transcriptional repressor of the *INK4/ARF* locus encoding p16^INK4A^ and p14^ARF^. Therefore, downregulation of E6AP in NSCLC results in the decrease in p16^INK4A^ expression. NSCLC patients with the characteristic of E6AP-low/CDC6-high/p16-low exhibit reduced overall survival. Furthermore, this characteristic is associated also with KRAS mutations in lung adenocarcinomas, implying a prognostic value of E6AP-low/CDC6-high/p16-low in the adenocarcinoma subtype of NSCLC [22].

### 2.5. Breast Cancer

E6AP acts as a transcriptional co-activator, promoting the transactivation by ER-α and PR, as well as AR [4]. E6AP could also ubiquitinate ER-α to target the receptor to proteasomal degradation [23]. In breast cancer, E6AP appears to play conflicting roles in promoting or suppressing cancer progression, which may be associated with its dual functions. E6AP expression is modestly downregulated in human invasive breast cancers, correlated with increased expression of ER-α and poorer prognosis of patients [23,54]. Transgenic mice that overexpress wild-type E6AP in mammary tissues fail to display any appreciable changes in morphology, while mice expressing a ligase-defective E6AP mutant develop mammary hyperplasia with concomitant upregulation of ER-α and PR [54]. These data suggest that, in mammary tissues, the E3 ligase activity of E6AP mostly plays a tumor-suppressive role by triggering degradation of the oncogenic drivers ER-α and PR, and this action of E6AP overshadows its potential oncogenic action as a co-activator. The significance of the control of ER-α levels at the degradation step is supported by another study showing that E6AP and calmodulin reciprocally control the stability of ER-α protein [55]. Breast cancer cell lines treated with a calmodulin antagonist, CGS9343B, show enhanced binding of E6AP to ER-α and accelerated degradation of the receptor [55]. In addition to the steroid receptors, a few breast-cancer-related oncoproteins have been identified to be substrates of E6AP-mediated ubiquitination. The steroid receptor co-activator oncogene AIB1 (amplified in breast cancer 1) is amplified and overexpressed in breast cancer [56,57,58]. Increased AIB1 expression is correlated with poor prognosis [59,60,61]. E6AP ubiquitinates and degrades AIB1 in breast cancer cell lines [62]. E6AP also ubiquitinates and degrades Enolase1 (ENO1), a glycolytic enzyme [63]. ENO1 expression is increased in breast cancer [64], which is likely to play a role in cancer-specific alterations in energy metabolism, i.e., Warburg effects, and also in metastasis via enzymatic degradation of extracellular matrix [65,66].

**Table 1:** E6AP Substrates in Cancer.

E6AP Substrate	Associated Disorder	Biological Role	References
PML	Burkitt’s Lymphoma, Prostate Cancer	Tumor suppressor, controls numerous proteins in PML-NB, induces cellular senescence	[20,32]
p27	Prostate Cancer	Cyclin-dependent kinase inhibitor, prevents progression from G_1_ to S phase	[21,67]
E2F1	NSCLC	Transcription factor, transactivates cell cycle genes including *CDC6*, whose product represses the *INK4/ARF* locus	[22]
ER-α, PR	Breast Cancer	Steroid receptors/transcription factors, drive expression of proliferative genes	[13]
ENO 1	Breast Cancer	Metabolic enzyme, controls energy metabolism and extracellular matrix degradation	[63]
AIBI	Breast Cancer	Oncoprotein, transcriptional co-activator of ER.	[57]
p53	HPV related cancers	Tumor suppressor, induces growth arrest and apoptosis	[19,68]
Clusterin	Prostate Cancer	Stress induced chaperone protein, tumor suppressor	[53]
HHR23A/RAD23A	Breast, Lung Cancer	Nucleotide excision repair protein	[69]

## 3. E6AP in Neurological Disorders

E6AP/UBE3A is widely known for its involvement in neurodevelopmental disorders. Genetic alterations in the *UBE3A* locus are linked with AS and chromosome 15q11.2–q13.3 duplication syndrome (Dup15q) [70]. Identifying the neuronal substrates of E6AP is important to elucidate the mechanism of neurological deficits in these disorders and develop effective treatments. Table 2 summarizes the E6AP targets that have been associated with neurological disorders.

### 3.1. Genetic Alterations in AS and ASD

**Figure 3:**
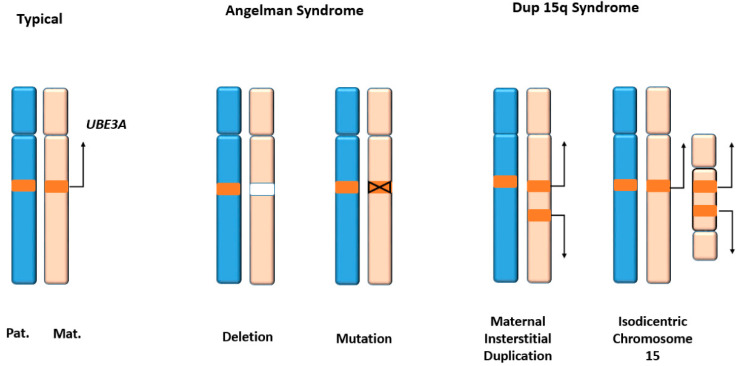
Genetic alterations in *UBE3A* locus. The *UBE3A* gene is located in chromosome 15 region q11.2-q13. Typically, only the maternal copy is expressed while the paternal copy is silenced in neurons. In Angelman syndrome (AS), the maternal allele is deleted or mutated. Multiple copies of *UBE3A* are expressed in Dup15q syndrome, which is characterized by autism spectrum disorder (ASD) phenotypes.

**Figure 4:**
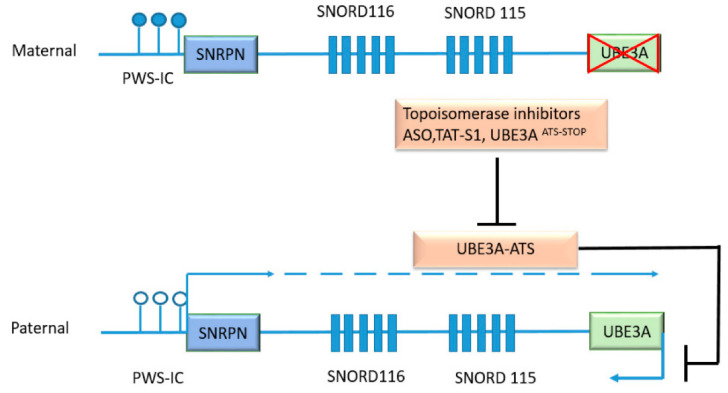
UBE3A-ATS as a mediator of genomic imprinting in neurons and as a therapeutic target. UBE3A-ATS is a large transcript which initiates at the Prader-Willi syndrome imprinting center (PWS-IC). The PWS-IC is not methylated (clear circles) in the paternal allele and allows the initiation of transcription. The progression of transcription through *UBE3A-ATS* locus is responsible for the paternal imprinting of UBE3A in neurons. *UBE3A-ATS* includes the protein-coding gene *SNRPN* and genes encoding small nucleolar RNAs *SNORD 116* and *SNORD 115*. Targeting UBE3A-ATS by topoisomerase inhibitors, antisense oligonucleotides, artificial transcription repressor TAT-S1, or insertion of stop codon *UBE3A*^ATS-STOP^ could restore UBE3A expression in AS neurons. The maternal PWS-IC is methylated (dark circles) and the expression of UBE3A-AS is silenced.

*UBE3A* gene dosage is critical for neuronal development. Loss of neuronal UBE3A expression results in AS, while increased dosage of the gene is associated tightly with autism spectrum disorders (ASD) (Figure 3). The *UBE3A* locus on chromosome region 15q11-q13.1 is paternally imprinted in neurons [70,71]. This is mediated at least partly by an antisense transcript UBE3A-ATS, which is paternally expressed and silences UBE3A expression (Figure 4) [72]. Consequently, UBE3A is expressed only from the maternal allele in normal neurons, and the loss of maternal UBE3A expression causes AS, which is characterized by a cheerful demeanor, developmental delay, impaired speech and seizures. On the other hand, increased copy numbers of *UBE3A* due to Dup15q markedly increase the penetrance of ASD [73] (Figure 3).

### 3.2. Role of E6AP in Neuron Morphology, Synaptic Plasticity and Excitability

E6AP orchestrates a broad range of effects on neuron morphology and functions. Abnormalities in neuron morphology are evident in AS. Abnormal dendritic spine length and polarization have been reported in AS model mice [74,75]. X-linked inhibitor of apoptosis (XIAP) has been recently identified as an E6AP target. XIAP is required for proper dendritic arborization. ASD-model transgenic mice overexpressing E6AP in neurons exhibit lower expression of neuronal XIAP, leading to caspase activation, microtubule degradation, and impaired spine maturation with less branching [76]. Learning and memory deficits are characteristic of AS. Altered synaptic plasticity and long-term potentiation (LTP) defects in hippocampal neurons have been found in AS model mice [77]. Decreased synaptic plasticity is observed in the visual cortex of mice with monocular deprivation [78,79]. The activity-regulated, cytoskeleton-associated protein (ARC), an E6AP target, controls synaptic function by promoting the internalization of α-amino-3-hydroxy-5-methyl-4-isoxazolepropionic acid (AMPA) receptors. Loss of E6AP expression in AS neurons results in an increase in ARC expression and a concomitant decrease in synaptic AMPA receptors, implying a mechanism of synaptic dysfunction in AS [80]. However, it is controversial whether ARC is actually a direct substrate of E6AP. Another suggested mechanism is that E6AP negatively regulates ARC at the transcriptional level [81]

The Small Conductance Potassium Channel (SK2) is also important for the induction of LTP and synaptic plasticity. SK2 has been shown to undergo E6AP-mediated ubiquitination. In response to N-methyl-D-aspartate (NMDA) receptor activation, SK2 channels are activated. E6AP ubiquitinates SK2 to promote internalization of the channel. *UBE3A*-deficient AS model mice show higher levels of SK2 in the hippocampal neurons and decreased synaptic plasticity [82]. Ephexin V, an established substrate of E6AP, is a guanine nucleotide exchange factor (GEF) which activates RhoA. Degradation of Ephexin V promotes excitatory synapse development. Elevated levels of Ephexin V in AS neurons may lead to defective synapse formation and cognitive impairment [83]. Epilepsy is present in 85% of AS patients [84]. Altered excitatory/inhibitory balance has been reported in AS model mice which display increased susceptibility to seizures [85,86]. Decreased GABA-ergic inhibitory input has been found in *UBE3A*-deficient AS model mice [87], and treatment with GABA agonists reduces the seizure susceptibility in those mice [88]. However, the E6AP targets that play key roles in controlling GABA signaling remain to be determined.

Sleep disturbances are reported in 75% of AS patients [89]. Brain and Muscle ARNT-Like 1 (BMAL1) is a clock protein critical to maintaining the circadian clock, and is a substrate of E6AP-mediated ubiquitination [90]. *UBE3A*-deficient mice have elevated levels of BMAL1 accompanied by impaired circadian rhythm [91].

It has been demonstrated that E6AP activates the Wnt signaling pathway with stabilization of β-catenin [92]. Wnt signaling is critical for normal development and is implicated in the pathogenesis of ASD [93,94]. TSC2, a negative regulator of mammalian target of rapamycin (mTOR), has been shown to be a E6AP substrate [95]. mTOR dysregulation has been described in AS and ASD [96].

Among the targets of E6AP that are controlled at the transcriptional level are the E3 ligase Ring1B [97]. It interacts with the polycomb group repressor complex and ubiquitinates histone H2A to impact on global gene expression. *UBE3A*-deficient mice exhibit increased levels of Ring1B and H2A in cerebellar purkinje neurons, suggesting its involvement in the development of AS neuronal deficits.

E6AP also activates the transcription of the *ESR2* gene encoding ER-β [98]. ESR2 is involved in brain development, while it also plays a neuroprotective role against neurodegenerative insults. The role of ESR2 in the development and progression of Alzheimer ’s disease (AD) has been well accepted [99,100]. ESR2 is important for synaptic plasticity and LTP via its regulation of brain-derived neurotrophic factor (BDNF) [101,102]. Overexpression of ESR2 in a rat model of AD reduced amyloid-β deposition in the hippocampus and improved the learning and memory of AD rats [99].

26S subunit, non-ATPase 4 (PSMD4), is a subunit of the 26S proteasome and an E6AP substrate [103]. The ubiquitination and degradation of PSMD4 may hamper proteasomal degradation of many cellular proteins and exert broad-spectrum effects on the proteostasis in neurons. A recent study showed that PSMD4 binds to the AZUL domain of E6AP and is necessary for its nuclear localization. This study suggested the nuclear form of E6AP plays an important role in neurodevelopment [104].

Xu et al., identified ALDH1A2, the rate-limiting enzyme of retinoic acid synthesis to be negatively regulated by E6AP. RA-mediated synaptic plasticity is altered with excessive *UBE3A* dosage. Administration of an RA antagonist or overexpression of UBE3A recapitulated the synaptic defects in ASD, while these defects were rescued by administration of RA [105].

A recent study identified phosphotyrosyl phosphatase activator (PTPA), an activator of protein phosphatase 2A (PP2A), as a substrate of E6AP-mediated ubiquitination [106]. Neurons in the AS model mice exhibit increases in PTPA levels and PP2A activity. Hemizygous knockout of the *Ptpa* gene or pharmacological inhibition of PP2A can ameliorate the defects in dendritic spine maturation of the AS model neurons, suggesting the significance of the E6AP-PTPA-PP2A pathway in AS pathophysiology.

**Table 2:** E6AP Substrates in Neurological Disorders.

Substrates	Associated Neurological Disorder	Biological Role	References
ARC	AS	Causes internalization of AMPA receptors at synapse. Important for synaptic plasticity	[80,81,107,108]
BMAL1	Tuberous Sclerosis	A clock protein critical to maintain the circadian clock under the control by the mTOR pathway	[90]
TSC2	Tuberous Sclerosis	Negative regulator of mTOR pathway, tumor suppressor.	[95,96]
Ring1B	AS	Ubiquitinates histone H2A. May affect global gene expression	[97]
ESR2/ER-β	Alzheimer’s Disease	Increase LTP and enhances synaptic strength	[98,109,110]
Ephexin V	AS	RhoA GEF, important for excitatory synapse development	[83]
Peroxiredoxin1	Alzheimer’s Disease	Antioxidant enzyme, protects from oxidative damage	[111,112,113,114,115]
SK2	AS, ASD	LTP and synaptic plasticity	[82,116]
XIAP	ASD	Required for dendritic arborization, suppresses caspase activation and microtubule degradation	[76,117,118,119]
PSMD4	AS	Subunit of 26S proteasome, controls global proteostasis. Interacts with the AZUL domain of UBE3A	[103]
β-catenin	ASD, AS	Transcription factor and canonical mediator of Wnt signaling	[92]
ALDH1A2	ASD	Rate limiting enzyme in RA synthesis	[105]
P18	ASD	Subunit of Ragulator complex	[120]
PTPA	AS	Activator of PP2A and controls dendritic spine morphology	[106]

## 4. Therapeutic Approaches to Target E6AP and Downstream Effectors

### 4.1. Challenges in Identifying HECT E3 Inhibitors

HECT E3 ligases are attractive therapeutic targets because of their involvement in a variety of human diseases. However, the efforts to develop small molecule inhibitors of HECT E3s are still in their infancy. The complexity of ubiquitination reactions and weak interactions between E3s and their substrates pose a challenge in identifying inhibitors of HECT E3s by high-throughput screens [121,122]. Small molecule screens have been performed successfully for classical targets such as kinases, proteases, G-protein coupled receptors (GPCRs), ion channels, and nuclear receptors, which typically possess well-defined small-molecule binding pockets. Several small-molecule inhibitors of RBR and RING E3 ligases have been published, including the inhibitors of HOIP, VHL, WWP2 and RNF4 [123,124,125,126,127,128,129]. However, small-molecule binding pockets in HECT E3s have been poorly defined. Detailed analysis of three-dimensional structures of the HECT domain and mutational analyses will facilitate the development of high-affinity, small-molecule modulators. Here, therapeutic strategies specific to E6AP are discussed, targeting its E3 ligase and co-activator functions.

### 4.2. Therapeutic Strategies for HPV-Induced Cancers

HPV-induced cancers are still highly prevalent. Although the HPV vaccines have been widely introduced, it will take decades for their preventive effects against cancers to become dominant. Thus, target-based therapies against HPV-induced cancers are still in critical need. Disruption of the E6AP–E6-p53 complex serves as an attractive therapeutic target, as it would ultimately re-activate p53, leading to growth arrest and apoptosis of HPV-transformed cells.

The crystal structure of E6AP-E6-p53 ternary complex was identified by Zapien et al. [130]. This structure provides a framework for the design of inhibitory therapeutic strategies against E6-E6AP and E6-p53 interfaces (Figure 1a). A recent study demonstrated that the binding with E6 causes a conformational change in E6AP structure, which facilitates the binding of p53 in close proximity to the catalytic center of E6AP [131], providing further insight into the mechanism of E6AP activation. There have been multiple small molecule approaches for targeting the E6-p53 and E6AP-E6 interfaces as well as for inhibiting the E3 ligase activity of E6AP, as discussed below.

Mutagenesis of critical residues at the E6–p53 interface abrogates p53 degradation. According to the structural model, p53 is bound on a cleft spanning both the N-terminal and C-terminal domains of E6 [130]. N-terminal residues of E6 that are located in the central region of the p53-binding cleft, i.e., Asp44, Phe47 and Asp49, play a critical role in this interaction and are highly conserved among high-risk mucosal HPV genotypes [130]. Given the importance of this region in HPV-induced oncogenesis, this interface has become an attractive target for small molecule development. Compound 12 has been identified as an inhibitor of the E6-p53 interaction that can reduce the viability and proliferation of HPV-positive cells [132].

The LxxLL-containing alpha helix of E6AP within the HECT domain binds to a hydrophobic binding groove in E6 [133]. This binding pocket offers a favorable niche for small molecules. Flavanoid compounds, Luteolin and CAF024, which mimic leucines in the conserved alpha helical motif of E6AP have been found to inhibit the E6–E6AP interaction [134]. Molecular modelling and simulation studies have revealed some additional inhibitors of the E6–E6AP interaction [135]. It has been demonstrated that the space between the two Zinc finger domains of E6 constitutes the LxxLL binding pocket [136]. Beerheide et al., identified compounds that eject Zn specifically from E6 and disrupt the E6–E6AP interaction. One such compound, 4,4′-dithiodimorpholine, reduced the viability of tumorigenic HPV cells with p53 upregulation.

Other approaches to inhibit E6 activity include peptides that interact with the E6AP-binding groove [137]. Intracellular antibodies or “Intrabodies” have also been shown to inhibit the growth of HPV-positive cancer cells [138]. However, the large size and complex structure of intracellular antibodies is a limitation in successful drug development [139].

Biochemical and structural analyses of E6AP have shown that the fully active form of E6AP is a trimer, and E6 oncoprotein promotes the trimerization. The interaction between the Phe727 residue in the HECT domain of E6AP and a hydrophobic pocket of E6 is critical to the formation of this trimer. N-acetyl phenylalanine blocks E6AP trimerization by substituting Phe727 and inhibits its E3 activity at a high concentration (Ki = 12 mM) [8]. Another approach using compounds mimicking natural products to inhibit the E3 ligase activity of E6AP has been described. Macrocyclic N-methyl peptide inhibitor, CM11-1, can inhibit E6AP activity to catalyze ubiquitination of Prx1 and p53 [9].

### 4.3. Targeting UBE3A in Neurodevelopmental Disorders

Current therapies for AS are only directed toward mitigating symptoms, such as anti-seizure medications and physical therapies. There have been multiple approaches to develop mechanism-based therapies for AS (Figure 4).

#### 4.3.1. Approaches to Target UBE3A-ATS

Efforts have been ongoing to reverse the paternal silencing of *UBE3A* in AS with mutations or deletions on the maternal allele. Several topoisomerase inhibitors, including topotecan, have been demonstrated to inhibit the transcription of the nuclear-localized long non-coding RNA Ube3A-ATS and allow the expression of *UBE3A* from the paternal allele [140]. Although the pleiotropic effects of topotecan that inhibit the transcription of many other synaptic genes is a concern [141,142], the observed effects of topoisomerase inhibitors on *UBE3A* regulation are encouraging for the future development of drugs that can unleash expression of the imprinted *UBE3A* allele.

Antisense oligonucleotides (ASO) offer an approach for gene therapies with high specificity, while the delivery of nucleotides remains challenging. ASO targeting Ube3a-ATS can effectively unsilence the paternal *UBE3A* allele in neurons, and ameliorate some cognitive deficits (e.g., “freezing”) in AS model mice. However, the ASO shows no effects on other behavioral defects [143]. Recently, the FDA granted the Orphan Drug designation for the UBE3A-ATS-targeting oligonucleotides to facilitate drug development.

An alternate approach to unsilence the paternal allele was to provide dietary supplements of methionine to increase methylation, thereby repressing the transcript which includes UBE3A ATS. However, two clinical trials with methionine supplementation reported no significant difference in the clinical outcome between the control and the treatment groups [144,145]. Forced activation of the *UBE3A* promoter or repression of UBE3A-ATS using artificial transcription factors is another feasible strategy. An artificial transcription factor, TAT-S1, which represses the locus encoding UBE3A-ATS, has been shown to increase Ube3a expression throughout the brain of AS model mice [146].

#### 4.3.2. Therapeutic Interventions of Downstream Effectors

Several experimental studies evaluated the effects of therapeutic interventions of effectors downstream of UBE3A in AS models. Reducing the expression levels of ARC alleviated audiogenic seizures in *Ube3a*-deficient mice, whereas there were no observed changes in motor deficit or ultrasonic vocalizations [108]. Excessive inhibitory phosphorylation of calmodulin-dependent protein kinase II (CAMKII) has been implicated for impaired synaptic plasticity in AS [147]. Levodopa can prevent the CAMKII phosphorylation and reduce the seizure propensity and deficits in motor performance, hippocampal learning and plasticity in the AS model mice [148]. A clinical trial of Levodopa for AS is on-going, and its data are awaited (NCT01281475). Ampakines, the modulators of AMPA receptors, have been shown to increase BDNF release and improve hippocampus-dependent learning behavior in AS model mice, suggesting their potential to alleviate LTP in AS. Blocking SK2 channels may also improve LTP, memory and learning behavior and restored activity dependent on actin polymerization. Systemic injection of a SK2 channel blocker has been shown to restore fear conditioning in the AS model [82,116,149]. The significance of the mTOR pathway in brain development and synaptic plasticity has been well established [96,150]. Hyperactivation of mTORC1 and hypoactivation of mTORC2 in *UBE3A*-deficient mice is apparently associated with motor dysfunction and deficits in LTP, fear conditioning and memory, which could be restored by the mTORC1 inhibitor Rapamycin as well as the mTORC2 activator A-443654 [151].

It has been shown that E6AP regulates mTORC1 signaling by targeting p18, a subunit of the Ragulator complex [120]. Hyperactivation of mTORC1 is also observed in ASD associated with mutations in genes upstream of mTOR, e.g., tuberous sclerosis complex (TSC), fragile X syndrome, and neurofibromatosis. Rapamycin has been shown to be effective for ASD associated with PTEN mutations, as well as for the ASD model mice [150,152].

## 5. Concluding Remarks

Therapeutic targeting of E3 ligases requires a multi-faceted approach including comprehensive profiling of the ubiquitination substrates, thorough analyses of the enzyme structures and efficient development of small molecule modulators. In this review, we have discussed the roles of E6AP/UBE3A in the pathophysiology of cancers and neurodevelopmental disorders, and highlighted the E6AP-substrate interactions and downstream pathways that could be therapeutic targets. With the development of novel substrate profiling technologies such as Orthogonal Ubiquitin Transfer [153], more information about previously undefined E6AP substrates is expected soon. While proteasome inhibitors are widely used in clinic [154,155,156,157], small molecules targeting E3 ligases are still in developmental or preclinical phases. Novel screening technologies will further build the avenue to therapeutic discoveries, such as activity-based probes, high-throughput crystallography, and sophisticated uses of mass spectrometry. In addition to inhibitors of E3 activities or protein–protein interactions, the promising techniques of targeted protein degradation, such as proteolysis targeting chimeras (PROTACs) and specific and nongenetic IAP-dependent protein erasers (SNIPER) [158,159,160], will widen the strategies to target the ubiquitination system in human diseases.
